# Effects of Montelukast on Arsenic-Induced Epithelial-Mesenchymal Transition and the Role of Reactive Oxygen Species Production in Human Bronchial Epithelial Cells

**DOI:** 10.3389/fphar.2022.877125

**Published:** 2022-04-19

**Authors:** Huang-Chi Chen, Hsin-Ying Clair Chiou, Mei-Lan Tsai, Szu-Chia Chen, Ming-Hong Lin, Tzu-Chun Chuang, Chih-Hsing Hung, Chao-Hung Kuo

**Affiliations:** ^1^ Department of Internal Medicine, Kaohsiung Municipal Siaogang Hospital, Kaohsiung Medical University, Kaohsiung, Taiwan; ^2^ Division of Pulmonary and Critical Care Medicine, Department of Internal Medicine, Kaohsiung Medical University Hospital, Kaohsiung Medical University, Kaohsiung, Taiwan; ^3^ Teaching and Research Center, Kaohsiung Municipal Siaogang Hospital, Kaohsiung Medical University, Kaohsiung, Taiwan; ^4^ Kaohsiung Medical University Hospital, Kaohsiung Medical University, Kaohsiung, Taiwan; ^5^ Graduate Institute of Medicine, College of Medicine, Kaohsiung Medical University, Kaohsiung, Taiwan; ^6^ Department of Pediatrics, School of Medicine, College of Medicine, Kaohsiung Medical University, Kaohsiung, Taiwan; ^7^ Division of Nephrology, Department of Internal Medicine, Kaohsiung Medical University Hospital, Kaohsiung Medical University, Kaohsiung, Taiwan; ^8^ Faculty of Medicine, College of Medicine, Kaohsiung Medical University, Kaohsiung, Taiwan; ^9^ Department of Microbiology and Immunology, School of Medicine, College of Medicine, Kaohsiung Medical University, Kaohsiung, Taiwan; ^10^ Department of Medical Research, Kaohsiung Medical University Hospital, Kaohsiung Medical University, Kaohsiung, Taiwan; ^11^ M.Sc. Program in Tropical Medicine, College of Medicine, Kaohsiung Medical University, Kaohsiung, Taiwan; ^12^ Department of Respiratory Therapy, College of Medicine, Kaohsiung Medical University, Kaohsiung, Taiwan; ^13^ Research Center for Environmental Medicine, Kaohsiung Medical University, Kaohsiung, Taiwan; ^14^ Department of Pediatrics, Kaohsiung Municipal Siaogang Hospital, Kaohsiung Medical University, Kaohsiung, Taiwan; ^15^ Division of Gastroenterology, Department of Internal Medicine, Kaohsiung Medical University Hospital, Kaohsiung, Taiwan

**Keywords:** montelukast, epithelial-mesenchymal transition, oxidative stress, bronchial epithelium, NF-κB

## Abstract

**Background:** Epithelial-mesenchymal transition (EMT) of airway lung epithelial cells is considered a major driver of fibrosis and airway remodeling. Arsenic exposure is well known to cause the malignant transformation of cells, including those in the lung. Accumulating studies have shown that arsenic exposure is associated with chronic pulmonary diseases. However, clinical treatment for arsenic-induced pulmonary damage has not been well investigated.

**Materials and Methods:** The therapeutic effects of montelukast and its combination with fluticasone on sodium arsenite-induced EMT changes in normal human bronchial cells were investigated. The cell migration ability was evaluated by Transwell and wound healing assays. EMT marker expression was determined by immunoblotting. Furthermore, the role of reactive oxygen species (ROS) generation in arsenic-induced EMT and the effect of montelukast on this process were determined by ROS inhibitor treatment and ROS measurement, respectively.

**Results:** Montelukast was effective at reducing arsenic-induced cell migration and mesenchymal protein (fibronectin, MMP-2, N-cadherin, β-catenin, and SMAD2/3) expression. Arsenic-induced ROS production was attenuated by pretreatment with montelukast. Treatment with the ROS inhibitor N-acetyl cysteine reduced arsenic-induced NF-kB phosphorylation and the mesenchymal protein expression, indicating that ROS production is critical for arsenic-induced EMT. In addition, combined treatment with montelukast and fluticasone reversed the inhibitory effects of montelukast on cell migration. The expression of fibronectin, MMP-2 induced by arsenic was further enhanced by the combination treatment compared with montelukast treatment only.

**Conclusion:** This study demonstrated that montelukast is effective at reducing arsenic-induced EMT in human bronchial epithelial cells. Through the inhibition of arsenic-induced ROS generation and NF-kB activation, which is critical for arsenic-induced EMT, montelukast inhibited arsenic-induced cell migration and the expression of extracellular matrix proteins and several EMT-regulating transcription factors. The combination of fluticasone with montelukast reversed the inhibitory effect of montelukast on arsenic-induced EMT. This study provides therapeutic strategies and mechanisms for arsenic-induced pulmonary epithelial damage.

## Introduction

Arsenic contamination is a major public health concern and threatens the health of millions of people worldwide. Previous studies have shown that chronic exposure to arsenic damages several organs, including the lung. Arsenic is classified as a group I carcinogen by the International Agency for Research on Cancer. The association between arsenic exposure and lung cancer has been well established (Straif et al., 2009). In addition, accumulating studies have shown that chronic arsenic exposure is positively correlated with the incidence of respiratory symptoms, chronic lung disease mortality (Sanchez et al., 2016), and lung function decrements (Parvez et al., 2013; Sanchez et al., 2018). Arsenic exposure is positively associated with nonmalignant lung diseases such as chronic obstructive pulmonary disease, bronchiectasis (Guha Mazumder, 2007), lung fibrosis (Assad et al., 2018; Wang P. et al., 2021; Xiao et al., 2021), and asthma (Siddique et al., 2020). Currently, the most popular options for the treatment of chronic arsenic overexposure include chelating and antioxidant therapies. However, the therapeutic effects, in terms of relieving lung symptoms caused by arsenic exposure, are inconsistent with chelating agents (Bjorklund et al., 2020). The usefulness of different therapies for arsenic-associated pulmonary diseases warrants further study.

Epithelial-mesenchymal transition (EMT) is characterized by loss of the epithelial phenotype and acquisition of the mesenchymal phenotype and is important in the regulation of not only the invasion and migration of tumor cells but also development and wound repair processes. The major events of EMT include the loss of cell–cell junctions and epithelial polarity, cytoskeletal reorganization, and an increase in the ability of the extracellular matrix (ECM) to regulate invasion (Kalluri and Weinberg, 2009). EMT is induced by extracellular factors/stimuli (Liu et al., 2010; Sohal et al., 2010) and is considered a universal process in lung diseases that promotes tissue remodeling (Hackett, 2012; Pain et al., 2014) and fibrosis (Jia and Kim, 2017; Rout-Pitt et al., 2018). Chronic exposure to arsenic induces neoplastic transformation of several tissues through the induction of cancer-related type III EMT (Jiang et al., 2013; Chang and Singh, 2019a; Tryndyak et al., 2020; Zhou et al., 2021). Moreover, arsenic was reported to perturb type I EMT in a chick embryo heart during development (Lencinas et al., 2010). In our previous studies, arsenic exposure at 10^–6^ M promoted cell migration and the expression of several mesenchymal proteins, especially ECM proteins, in human bronchial epithelial cells. In addition, several studies have demonstrated that sodium arsenite exposure could induce pulmonary fibrosis *in vitro* and *in vivo* (Dai et al., 2019; Wang P. et al., 2021; Xiao et al., 2021). These results provide evidence that EMT may be the critical mechanism by which arsenic induces pulmonary diseases.

Montelukast is a cysteinyl leukotriene receptor (CysLT1R) antagonist that was approved as an effective therapeutic agent for asthma as well as allergic rhinitis. It was reported to have antitumor activity through the induction of apoptosis and growth arrest (Zovko et al., 2018). It was also found to inhibit cell migration and invasion by inhibiting MMP-2 and MMP-9 expression in human glioblastoma cells (Piromkraipak et al., 2018). CysLT1R antagonists have been demonstrated to suppress EMT and fibrotic protein expression. Montelukast (10^–4^ M) suppresses EMT induced by eosinophils through the inhibition of TGF-β signaling (Hosoki et al., 2014). The expression of profibrotic cytokines, including IL-6, IL-10, IL-13, and TGF-β, was inhibited by montelukast in a bleomycin-induced pulmonary fibrosis mouse model (Shimbori et al., 2011). Pranlukast was shown to reduce airway remodeling through the inhibition of TGF-β/Smad signaling in an OVA-sensitized and challenged asthma mouse model (Hur et al., 2018).

In this study, we evaluated the therapeutic potential and mechanism of montelukast in treating arsenic-induced lung damage using normal human bronchial epithelial cells. The effects of montelukast on arsenic-induced EMT and ROS generation was examined. Furthermore, the combination of montelukast with fluticasone was assessed.

## Materials and Methods

### Cell Culture, Drugs, and Reagents

Normal human bronchial epithelial cells (NHBE, Lonza) were maintained in Keratinocyte SFM basal medium supplemented with 5 μg/L human recombinant epithelial growth factor (EGF), 50 mg/L bovine pituitary extract (BPE), 5 mg/L insulin, and 25 nM hydrocortisone at 37°C in a 5% CO_2_ atmosphere. Cells were passaged every 3 days and plated for experimental treatment within 6 passages.

NaAsO_2_, montelukast, fluticasone, N-acetylcysteine, and BAY117082 were purchased from Sigma–Aldrich (United States). Antibodies against fibronectin (Sigma, F3648), MMP-2 (GeneTex, GTX 104577), GAPDH (GeneTex, GTX100118), SMAD2/3 (GeneTex, GTX111123), β-catenin (R&D, AF1329), N-Cadherin (GeneTex, GTX127345), and E-Cadherin (GeneTex, GTX100443) were used.

### Western Blotting

Cells were pretreated with drugs or inhibitors (montelukast, fluticasone, BAY117082, NAC) for 2 h, and then combined treated with sodium arsenite for additional 24 h. After treatments, the cells were washed twice with ice-cold PBS and lysed with RIPA buffer containing protease inhibitor (cOmplete™, Mini, EDTA-free Protease Inhibitor Cocktail, Sigma–Aldrich) and phosphatase inhibitor (PhosStop™, Sigma–Aldrich). The protein lysate was harvested by centrifugation at 14,000 rpm at 4°C for 15 min to pellet the cell debris. The protein concentration was determined using the BCA Dual-Range BCA Protein Assay Kit (Visual Protein, Taiwan). A total of 10 µg of protein lysate was separated by 6 ∼ 13% SDS–PAGE and transferred onto PVDF membranes (Millipore, United States). After blocking with 5% skim milk at room temperature for 1 h, the membranes were incubated with primary antibody at 4°C overnight. After three washes with 0.1% TBST, the membrane was incubated with HRP-conjugated anti-rabbit IgG or anti-goat secondary antibody (Jackson ImmunoResearch Laboratories, West Grove, PA). The membrane was developed by reacting with chemiluminescence HRP substrate (Merck, Germany), and the protein bands were visualized and captured using a ChemiDoc-It 810 Imager (Ultra-Violet Products, United States). The protein bands were quantified using ImageJ.

### DCFH-DA Staining of Total ROS

The staining protocol was performed as described (Li et al., 2013). HBE cells were seeded on a dark, clear bottom 96-well microplate with 15,000 cells per well. The cells were pretreated with montelukast for 2 h and then treated with NaAsO_2_ for 3 h. The cells were then stained with 10 µM DCFH-DA at 37°C for 30 min by added the dye into the well. After DCFH-DA staining, the cells were washed with PBS and immediately assessed on a fluorescence microplate reader (Ex/Em = 485/535 nm). The result was represented as a subtraction of the tested well and the unstained well. Fluorescence was also observed under a fluorescence microscope (Leica).

### Wound Healing Assay

NHBE cells were plated in 12-well plates and allowed to grow to confluence. Inhibitors were pretreated 2 h before arsenic treatment. After arsenic treatment for 24 h, wounds were made with 200-µl tips and washed twice with PBS to remove the floating cells. NaAsO_2_ was added in the presence of inhibitors and incubated for analysis at 6, 9, 12 h as indicated. Images were captured using a microscope (magnification, ×200; Olympus, Tokyo, Japan), and the wound area was analyzed by ImageJ. The wound area was normalized to that at 0 h as 100% and expressed as wound coverage%. The calculation was performed as [(wound area_0h_ –wound area_after incubation_)/wound area_0h_]x100%.

### Transwell Migration Assay

NHBE cells were exposed to asthma drugs for 2 h, followed by NaAsO_2_ exposure for 24 h. The cells were then detached and replated onto the upper chamber of a Transwell with 8-μm pores (BD Biosciences, Franklin Lakes, NJ, United States) at a density of 5 × 10^4^ cells in 200 µl of supplement-free Keratinocyte SFM. The lower chamber was filled with Keratinocyte SFM containing 100 ng/ml EGF, 50 mg/L bovine pituitary extract (BPE), 5 mg/L insulin, and 25 nM hydrocortisone. After incubation, the cells were fixed with methanol for 10 min at room temperature followed by staining with 0.2% crystal violet for 10 min at room temperature. All cells on the membrane were imaged and counted to ensure that an equal number of cells were seeded. After that, the cells in the upper side of the chamber were removed with a cotton swab, and the migrated cells on the bottom side of the membrane were imaged for quantitative analysis. Five randomly selected areas (×200) were selected under a light microscope. The cell migration activity was quantitated by determining the occupied area *via* ImageJ and applying the following formula: (the bottom side cell occupied area/total cell occupied area).

### Cell Morphology Analysis

Cell morphology change was analyzed by radius-ratio method (Ren et al., 2015). Briefly, NHBE cells were pretreated with montelukast for 2 h, and followed by combined treatment with sodium arsenite for another 48 h. The cell images of each group were observed in ×200 magnification and acquired using Leica Flexacam C1 (Leica microsystems, Germany). The radius ratio was determined using Ratio between max radius and min radius of the cells. Total of 60 cells were analyzed in each group for statistical analysis.

### Statistical Analysis

The unpaired Student t-test and one way ANOVA test were used for the statistical analysis. Values are presented as means ± S.E.M. Statistical signifcance was determined as *p* < 0.05.

## Results

### Montelukast Inhibits the Arsenic-Induced Migration of Human Bronchial Epithelial Cells

**FIGURE 1:**
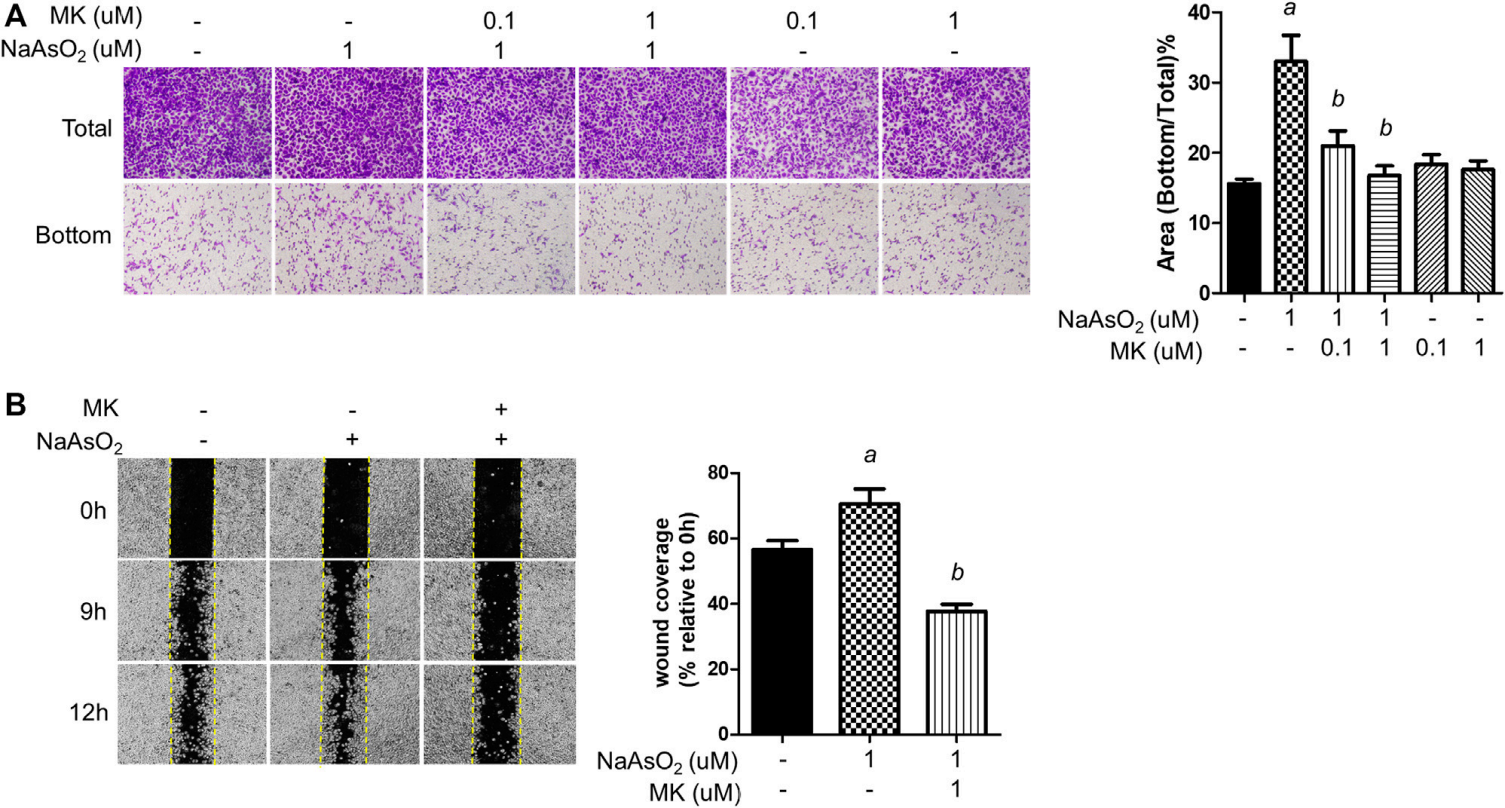
The effect of montelukast on the arsenic-induced migration of NHBE cells. **(A)** Cells were treated with montelukast (MK, 0.1 and 1 µM) 2 h prior to NaAsO_2_ stimulation followed by combined treatment for 24 h. The cells were subjected to a Transwell migration assay. Twenty-seven hours later, the cells were fixed and stained with crystal violet. The representative image (left panel) and the quantitative results (right panel) are shown. **(B)** The cells were pretreated with 1 µM montelukast for 2 h and then arsenic was added. After 24 h, a wound was made, and images were taken at different time points as indicated. The representative image (left panel) and the quantitative results (right panel) are shown. *a*: *p* < 0.05 compared with the vehicle control group. *b*: *p* < 0.05 compared with the NaAsO_2_ group.

Cell migration is a critical property of EMT. The effects of montelukast on arsenic-induced cell migration were determined by Transwell and wound healing assays in human bronchial epithelial (NHBE) cells. In the Transwell migration assay, cell migration was increased upon 1 µM NaAsO_2_ treatment. Pretreatment with montelukast at 0.1 and 1 µM had inhibitory effects on arsenic-induced cell migration compared with treatment with arsenic alone (Figure 1A) In the wound healing assay, arsenic treatment increased wound closure compared with untreated cells. Pretreatment with montelukast reduced the migration ability, as shown by reduced wound coverage (Figure 1B). Treatment with montelukast alone did not alter the migration ability of NHBE cells (Figure 1A). These results demonstrated that montelukast was effective for reducing arsenic-induced cell migration.

### Montelukast Reduces Arsenic-Induced Mesenchymal Protein Expression

**FIGURE 2:**
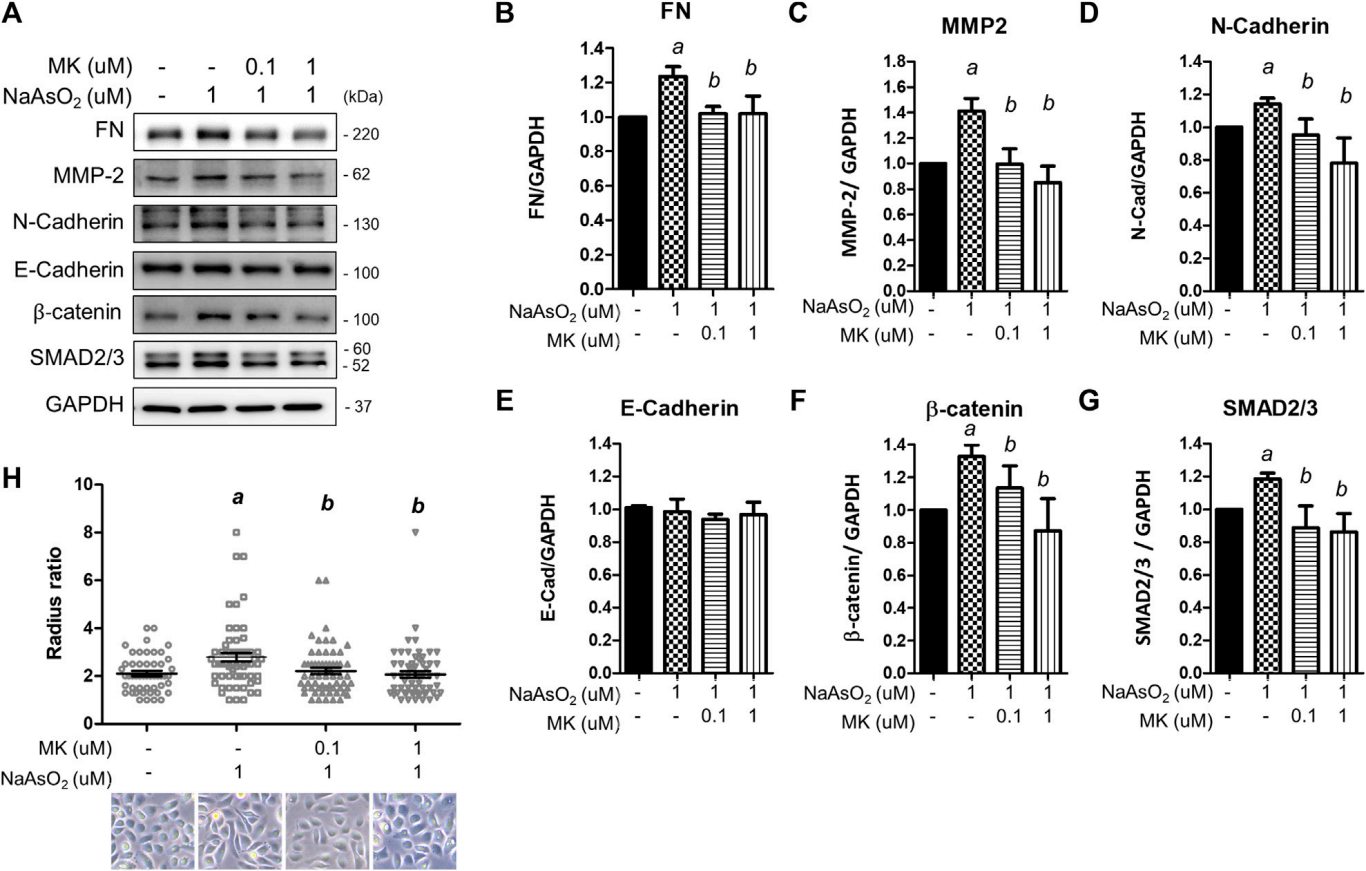
The effect of montelukast on arsenic-induced EMT marker expression in NHBE cells. Cells were pretreated with montelukast for 2 h, followed by combined treatment with 1 µM NaAsO_2_. After 24 h of treatment, the cells were harvested for protein expression analysis by western blotting. **(A)** Representative western blot images. **(B–G)** The quantitative results of protein expression changes are shown in **(C–G)**. *a*: *p* < 0.05 compared with the vehicle control group. *b*: *p* < 0.05 compared with the NaAsO_2_ group. **(H)** The cell morphology changes. NHBE cells were pretreated with 0.1 or 1 µM montelukast respectively for 2 h and followed by sodium arsenite for another 48 h. The cell morphology was analyzed by radius-ratio methods. *a*: *p* < 0.05 compared with the vehicle control group. *b*: *p* < 0.05 compared with the NaAsO_2_ group.

To examine the effects of arsenic on EMT marker expression, NHBE cells were treated with NaAsO_2_ for 24 h, and the protein lysate was harvested for western blot analysis. Arsenic treatment significantly induced the expression of several mesenchymal markers, such as extracellular matrix-regulating proteins [fibronectin and matrix metalloproteinase 2 (MMP-2)], cytoskeletal protein (N-cadherin), and transcription factors (β-catenin and SMAD2/3). The expression of E-cadherin was not altered by arsenic. The dose-response effects of arsenic on EMT marker expressions were performed. The results show that 1 uM of arsenic most significantly increased the mesenchymal proteins expressions (Supplementary Figures S1A-E). Moreover, 1 μM of NaAsO_2_ equals 75 ppb of arsenic, which is about the environmental relevant concentration in the drinking water of arsenic contaminated region (>50 ppb) (Smith et al., 2000). Thus, we had treated with 1 μM of sodium arsenite for 24 h on NHBE cells to evaluate the effect of montelukast on EMT. Montelukast treatment from 10^–8^∼10^−6^ M caused no significant changes on cell viability (Supplementary Figure S1G). Pretreatment with montelukast at 0.1 and 1 µM was effective in reducing the expression of these mesenchymal proteins (Figures 2A–G). Moreover, the treatment of 0.1 and 1 μM of montelukast alone or in combination with arsenic do not affect cell viability (Supplementary Figures S1G,H). Consistent with the mesenchymal protein expressions, arsenic induced morphological changes of the cell. The cells morphology become spindle shape after sodium arsenite treatment compared to vehicle control. Arsenic treatment significantly increased the radius-ratio of NHBE cells, which was reversed by pretreatment of montelukast (Figure 2H).

### Montelukast Reduces ROS Generation to Regulate Arsenic-Induced EMT

Oxidative stress is a widely accepted mechanism of arsenic toxicity (Hu et al., 2020). Here, we aimed to elucidate whether montelukast regulates arsenic-induced ROS generation, which is reported to be associated with EMT progression (Jiang et al., 2017).

**FIGURE 3:**
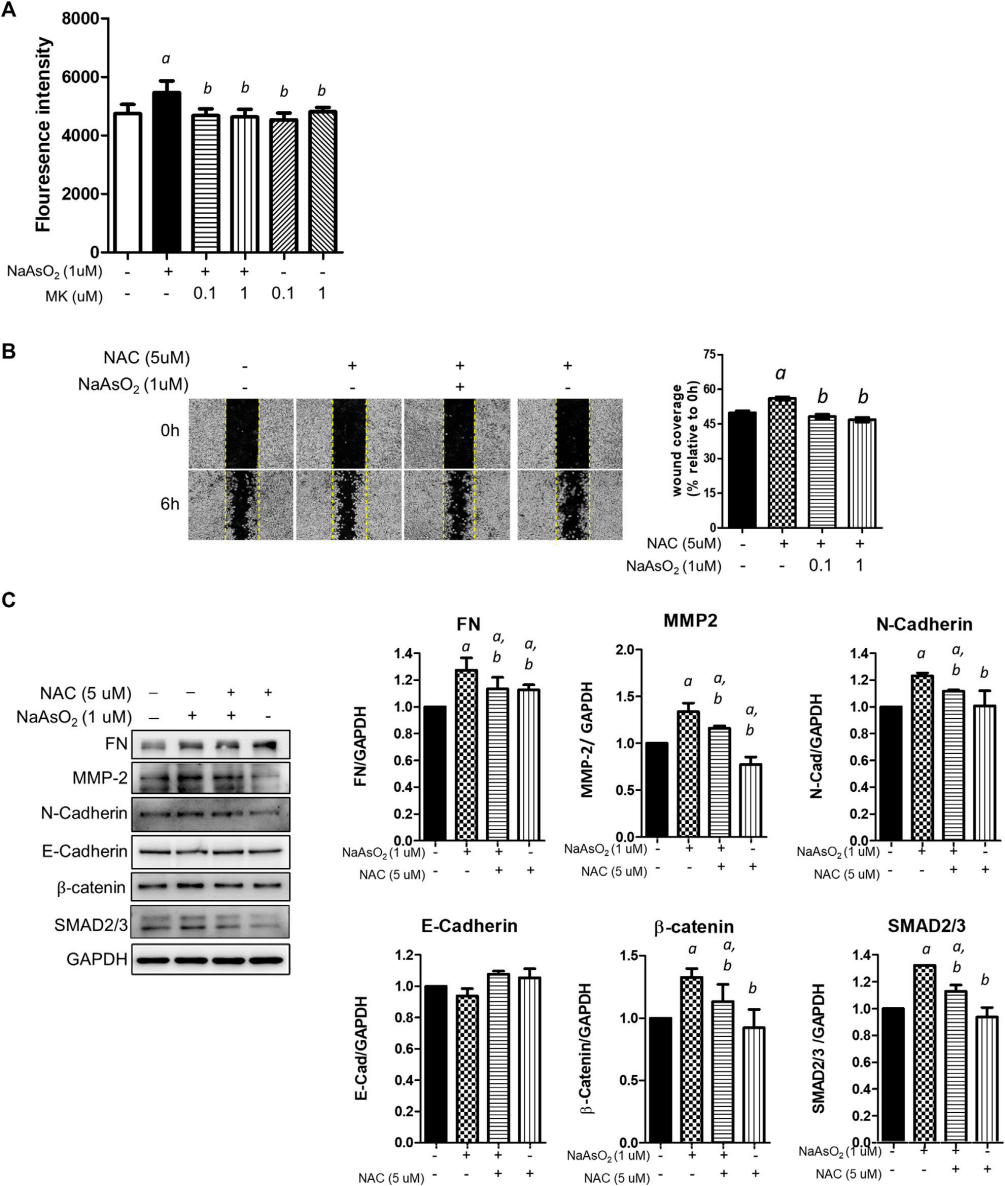
The effects of montelukast on ROS generation and of the inhibition of ROS generation on arsenic-induced EMT of NHBE cells. **(A)** Cells seeded in 96-well plates were pretreated with montelukast at 0.1 and 1 µM for 2 h, and then 1 µM NaAsO_2_ was applied for combination treatment. After 3 h of treatment, the cells were stained with 10 µM DCFH-DA for 30 min. After washing out the residue dye, the fluorescent signal was measured immediately. The quantitative results (left panel) and representative images (right panel) are shown. a: *p* < 0.05 compared with the vehicle control group. b: *p* < 0.05 compared with the NaAsO2 group. **(B)** Cells were pretreated with NAC for 2 h, and then 1 µM NaAsO2 for another 24 h. A wound was made with a 200-µl tip, and the wound coverage was analyzed 6 h after the wound was made. a: *p* < 0.05 compared with the vehicle control group. b: *p* < 0.05 compared with the NaAsO2 group. **(C)** Cells were pretreated with NAC for 2 h, and then 1 µM NaAsO_2_ was added for another 24 h. The cells were harvested for protein expression analysis by western blotting. *a*: *p* < 0.05 compared with the vehicle control group. *b*: *p* < 0.05 compared with the NaAsO_2_ group.

First, the effect of montelukast on arsenic-induced ROS generation was examined. NHBE cells were treated with arsenic and montelukast alone or in combination for 3 h, and ROS generation was determined by staining with DCFH-DA followed by quantitation of the fluorescence intensity of each well with a plate reader. The DCFH-DA was known to react with H_2_O_2_, ONOO^−^, lipid hydroperoxide and O_2_^−^, and was considered a general indicator for total ROS generation. The results indicated that arsenic treatment increased the fluorescence intensity compared with that in control cells (Figure 3A). The cells pretreated with montelukast showed reduced arsenic-induced ROS generation compared with those treated with arsenic only. Representative images showing the reduced fluorescent signal induced by montelukast are shown in the right panel (Figure 3A). These results demonstrated that montelukast reduced the ROS generation induced by arsenic in NHBE cells.

Moreover, to determine the role of ROS generation in the EMT of NHBE cells, we pretreated cells with the ROS scavenger N-acetylcysteine (NAC), and the effects on arsenic-induced EMT were examined. Pretreatment with NAC significantly inhibited arsenic-induced wound closure (Figure 3B) and reduced the expression levels of fibronectin, matrix metalloproteinase-2 (MMP-2), N-cadherin, β-catenin, SMAD2/3, and SNAIL, which were increased with arsenic treatment (Figure 3C). These results demonstrated that the induction of oxidative stress is involved in the arsenic-induced EMT of NHBE cells. Altogether, these results suggested that montelukast-mediated inhibition of ROS generation is critical for arsenic-induced EMT in NHBE cells.

### Montelukast Inhibits the Activation of NF-κB to Regulate Arsenic-Induced EMT

**FIGURE 4:**
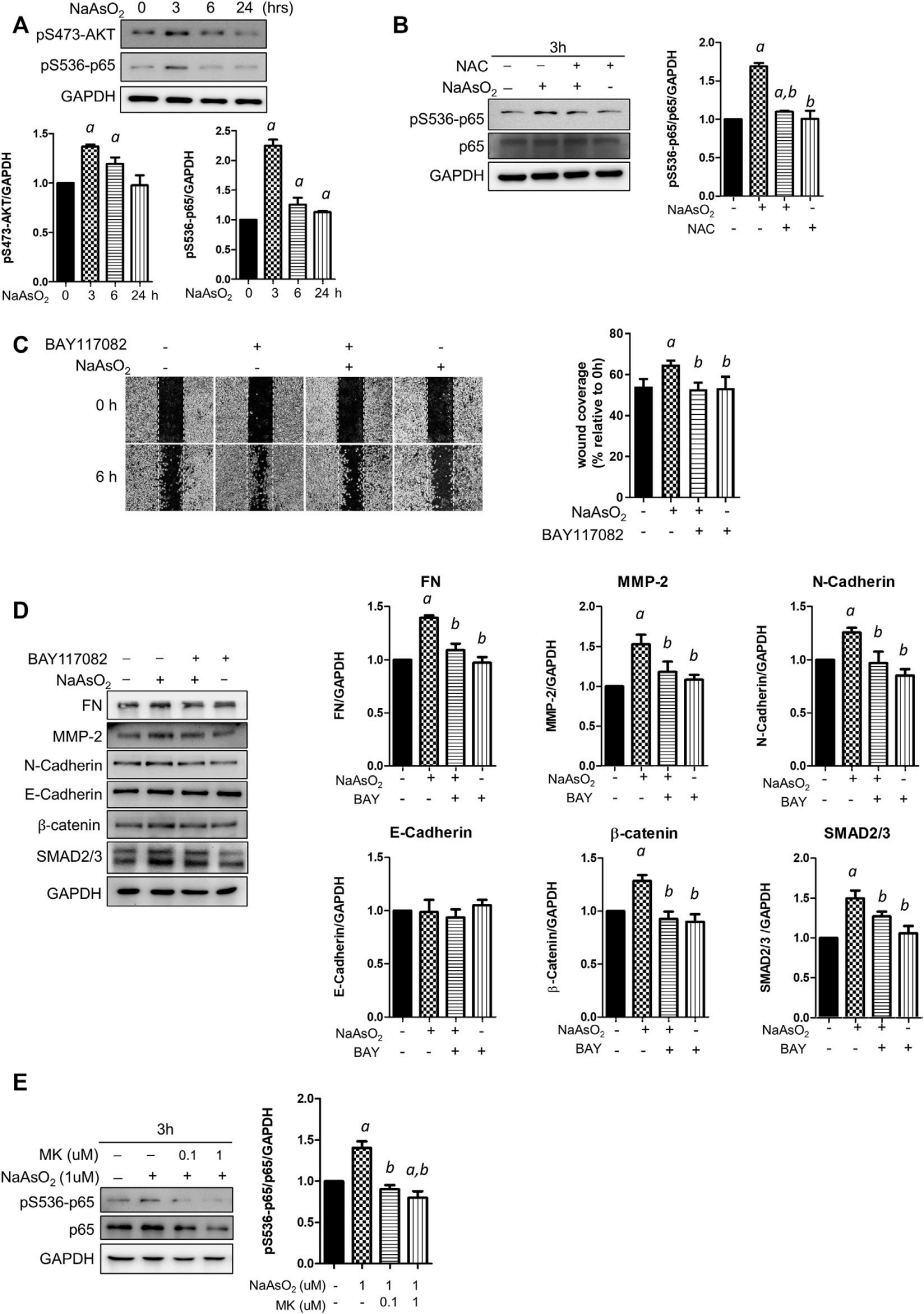
The effects of montelukast on NF-κB activation and of the inhibition of NF-κB on mediated arsenic-induced EMT of NHBE cells. **(A)** Arsenic induced the phosphorylation of AKT and p65. Cells were treated with 1 µM NaAsO_2_ for different time intervals, and the cells were harvested and subjected to western blot analysis using a phospho-antibody. **(B)** NAC treatment inhibited arsenic-induced phospho-p65. Cells were pretreated with NAC for 2 h, and then NaAsO_2_ was added for another 3 h. The expression of phospho-p65 was determined with a phospho-Ser536-p65 antibody. GAPDH served as the internal control. **(C)** Treatment with an NF-κB inhibitor reduced arsenic-induced cell migration. Cells were pretreated with 2 µM BAY117082 for 2 h and then treated with 1 µM NaAsO_2_ for another 24 h. A wound was made with a 200-µl tip, and the wound coverage was analyzed by comparing the cell-occupied area at 0 h versus that 6 h after the wound was made. The representative image (left panel) and the quantitative results (right panel) are shown. *a*: *p* < 0.05 compared with the vehicle control group. *b*: *p* < 0.05 compared with the NaAsO_2_ group. **(D)** Treatment with an NF-κB inhibitor reduced arsenic-induced mesenchymal marker expression. Cells were pretreated with 2 µM BAY117082 for 2 h, and then 1 µM NaAsO_2_ was added for another 24 h. The cells were harvested for protein expression analysis by western blotting. **(E)** Montelukast inhibited arsenic-induced phospho-p65 expression. Cells were pretreated with montelukast for 2 h, and then arsenic was added for another 3 h. The cells were harvested for protein expression analysis by western blotting. GAPDH served as the internal control.

To explore the downstream molecules mediating arsenic-ROS signaling for EMT regulation, the involvement of AKT-NF-κB, which is known as critical signaling for EMT regulation, was examined. Cells were treated with NaAsO_2_ for 3, 6, and 24 h, and the results showed that the phosphorylation of AKT and the NF-κB subunit p65 was induced at 3 h and then decreased at 24 h after NaAsO_2_ treatment (Figure 4A). The arsenic-induced phosphor-Ser536-p65 is inhibited by pre-treatment of NAC (Figure 4B), indicating that arsenic induced NF-kB activation through ROS generation.

To elucidate the role of NF-κB activation in arsenic-induced EMT, the cells were pretreated with the NF-κB inhibitor BAY117082, and cell migration and mesenchymal protein expression were analyzed. The results indicated that wound coverage was significantly induced by arsenic, which was reversed by combined treatment with BAY117082 (Figure 4C). The mesenchymal protein expression induced by arsenic was reversed by pretreatment with BAY117082, including fibronectin, MMP-2, N-cadherin, β-catenin, and SMAD2/3 (Figure 4D). These results indicate that NF-κB is an important mediator of arsenic-induced cell migration in NHBE cells.

To elucidate whether montelukast inhibits arsenic-induced EMT through the regulation of NF-κB activation, the effects of montelukast on arsenic-induced Ser536 phosphorylation of p65 were determined by western blotting. The results indicated that treatment with montelukast significantly reduced arsenic-induced phospho-Ser536-p65 expression (Figure 4E). These results indicated that montelukast inhibits arsenic-induced EMT through the regulation of NF-κB activation.

### Effects of the Combination of Montelukast and Fluticasone on Cell Migration and Mesenchymal Protein Expression

Since montelukast is frequently used in combination with fluticasone in the clinic, the effect of the combination of montelukast and fluticasone on arsenic-induced EMT in NHBE cells was examined. Considering the plasma concentration in human (average ∼ 0.2 μM within 8 h after single oral dose) (Balani et al., 1997), we had chosen the lower concentration of montelukast (0.1 μM) to combined with fluticasone.

**FIGURE 5:**
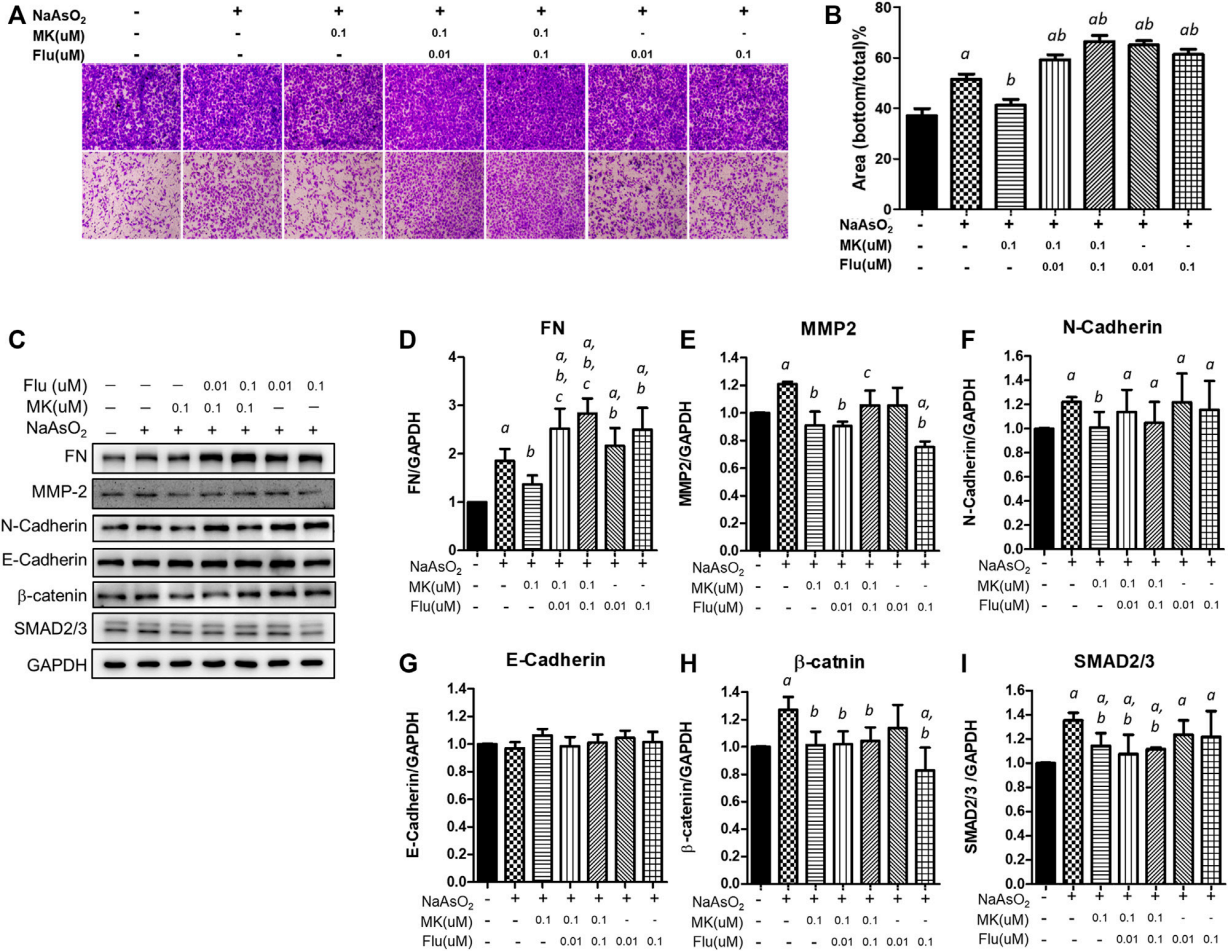
Effect of the combination of montelukast and fluticasone on NaAsO_2_-induced cell migration of NHBE cells. Cells were pretreated with medications for 2 h and then arsenic was added. **(A,B)** After 24 h of incubation, the cells were trypsinized, plated onto the inner side of the Transwell chamber and incubated for 36 h. The migrated cells were analyzed by the ratio of the bottom/total of the cell occupied area. Representative images (left panel) and the quantitative results are shown (right panel). **(C–I)** After 24 of incubation, the protein lysates were extracted and applied to western blot analysis. The representative image was shown in **(C)**, and the quantitative results were shown, respectively **(D–I)**. *a*: *p* < 0.05 compared with the vehicle control group. *b*: *p* < 0.05 compared with the NaAsO_2_ group. *c*: *p* < 0.05 compared with the montelukast plus NaAsO_2_ group.

Consistently, treatment with 0.1 µM montelukast reduced cell migration in the Transwell migration assay. However, the combination of montelukast with fluticasone at either 0.01 µM or 0.1 µM reversed the inhibitory effects of montelukast on cell migration (Figures 5A,B). Treatment of fluticasone show to increase the arsenic-induced cell migration. For the mesenchymal protein expressions, montelukast treatment consistently inhibit the arsenic-induced mesenchymal protein expressions. Fluticasone treatment increased the arsenic-induced fibronectin expressions but the arsenic-induced MMP-2, N-cadherin, b-catenin, and SNAD2/3 were not changed. For the combine treatment with montelukast and fluticasone, the effects of montelukast on inhibiting arsenic-induced fibronectin (FN) and MMP-2 expression was reversed, and the N-cadherin, β-catenin, SMAD2/3 expressions were not changed (Figures 5C–I). This result suggested that the addition of fluticasone blocks the inhibitory effect of montelukast on arsenic-induced EMT, possibly by reversing the expression of fibronectin.

## Discussion

Arsenic exposure causes both malignant and nonmalignant transformation of the pulmonary system and threatens the health of millions of people around the world. In this study, we examined the therapeutic effects of the clinical drug montelukast on arsenic-induced bronchial epithelial changes *in vitro*. We have demonstrated that through the inhibition of ROS generation and downstream AKT-NF-κB signaling activation, montelukast inhibits arsenic-induced EMT in human bronchial epithelial cells. These results provide a possible effective therapeutic agent for arsenic-induced pulmonary malfunctions.

EMT is a critical mechanistic change that contributes to tissue fibrosis, airway remodeling, and carcinogenesis. The major changes in epithelial cells undergoing EMT include 1) diminished cell junctions and loss of apical-basal cell polarity, 2) cytoskeletal remodeling and acquisition of cell motility, and 3) ECM remodeling and deposition. Several EMT changes were reported to be affected by arsenic. Arsenic trioxide (40 μM, 12 h) treatment induced tight junction damage to intestinal epithelial cells, which resulted in the downregulation of claudin-1 and claudin-5 (Jeong et al., 2017). The excess production of ECM caused by EMT contributes to fibrotic disorders. Arsenic exposure causes fibrotic-related changes in several organs, such as the kidney (Chang and Singh, 2019b), lung (Wang P. et al., 2021; Xiao et al., 2021), and liver (Wu et al., 2008). These studies demonstrated that arsenic significantly induced the expression of markers for fibrosis, such as fibronectin, collagen I, transforming growth factor β, and α-smooth muscle actin. These studies are consistent with our study: using normal human bronchial epithelial cells, we observed that sodium arsenite (1 μM, 24 h) promotes cell migration and the expression of mesenchymal proteins, including the adherent junction protein N-cadherin, and extracellular matrix-regulating proteins, including fibronectin and MMP-2. However, the epithelial marker protein E-cadherin was not significantly altered under this condition. Downregulation of E-cadherin has been considered as the hallmark of EMT. However, some studies have also found that E-Cadherin expression is preserved in several types of cancer cells that undergo EMT, and downregulation of E-Cadherin is not sufficient to induce EMT (Hollestelle et al., 2013; Chen et al., 2020; Nilsson et al., 2014; Wong et al., 2018). Holestelle’s study has reported that E-Cadherin was downregulated by the EMT process itself *via* downstream epigenetic silencing. Thus, it is suggested that E-Cadherin downregulation may not be necessary for the development and progression of arsenic-induced EMT. Moreover, for junctional proteins such as occludin and ZO-1, there were no significant expression changes with treatment (data not shown). It is suggested that our cell culture model does not include well-differentiated epithelial cells to represent an intact functional junction structure. This issue can be further addressed by using an air-liquid interface culture system, which has been widely accepted for assessing epithelial junction integrity.

Montelukast is a leukotriene receptor antagonist that acts as both a bronchodilator and an anti-inflammatory agent to ameliorate asthma symptoms. The activity of montelukast in EMT regulation has been reported. In a rat model of bronchopulmonary dysplasia (BPD), montelukast treatment inhibited the expression of hyperoxia-induced mesenchymal markers, such as collagen I (Col I), metalloproteinase-1 (MMP-1), MMP-3, TGF-β1, and Smad3, in lung tissues by inactivating the TGF-β1/Smad signaling pathway (Chen et al., 2020). Consistently, in this study, we showed that montelukast downregulates SMAD2/3 signaling to suppress arsenic-induced EMT. Moreover, we demonstrated that montelukast suppresses arsenic-induced β-catenin expression, which is a key effector of the WNT signaling pathway known to regulate EMT. In a meta-analysis, arsenic was reported to upregulate the WNT/β-catenin signaling pathway in normal cells and downregulate it in cancer cells. Arsenic at concentrations ranging from 0 to 7.5 µM has been demonstrated to have a dose-dependent effect on WNT/β-catenin signaling (Li and Ren, 2020). This is consistent with our results that arsenic (1 μM, 24 h) upregulates β-catenin expression. These results suggested that montelukast reduced arsenic-induced EMT through the suppression of multiple EMT-promoting pathways, including TGFβ/SMAD and WNT/β-catenin. Since TGFβ/SMAD and WNT/β-catenin pathways were also critical for type III EMT in the regulation of tumorigenesis, it is suggested that montelukast may have the potential for the prevention or therapy of lung cancer.

**FIGURE 6:**
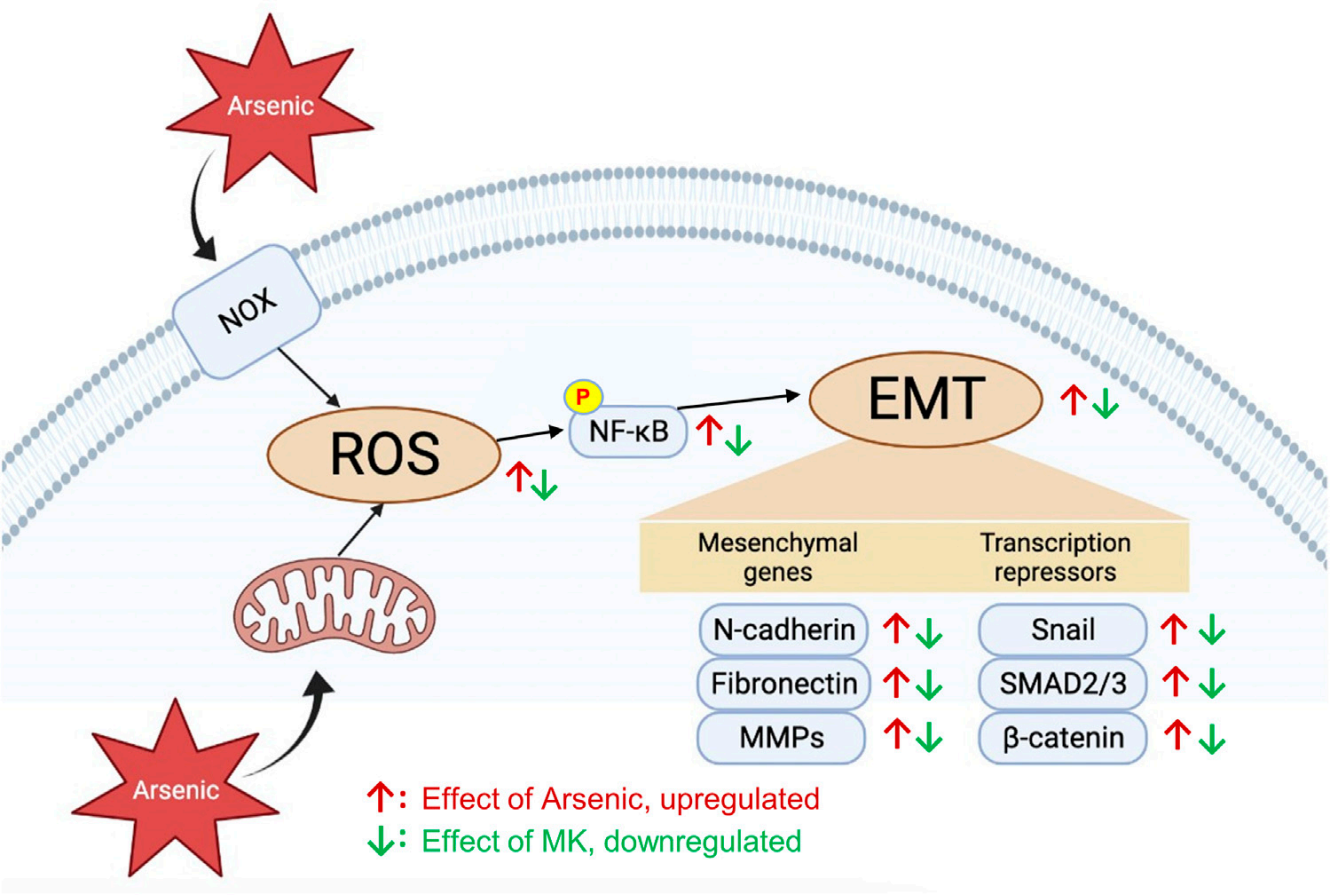
The effects and signaling molecules involved in montelukast-mediated attenuation of arsenic-induced EMT changes in human bronchial epithelial cells.

While the toxicity mechanisms of arsenic are complex and not fully understood, ROS generation is considered a common pathway in arsenic toxicity. ROS are important cellular secondary messengers that mediated diverse biological processes through the regulation of signaling pathways involved in cell proliferation, apoptosis, cell migration, and inflammation. Arsenic induces ROS generation in several ways: 1) activation of mitochondrial complexes I and III in the electron transport chain to increase O_2_- production, 2) activation of nicotinamide adenine dinucleotide phosphate oxidase (Nox), 3) the process of arsenic metabolism, 4) activation of the endoplasmic reticulum by trivalent dimethylarsinic acid, and 5) indirect induction of ROS generation by interfering with antioxidants (Hu et al., 2020). Excess ROS generation has been demonstrated to promote bronchial hyperresponsiveness (Jiang et al., 2014) and EMT progression (Jiang et al., 2017). Montelukast has been previously demonstrated to have powerful anti-ROS activity for H_2_O_2_-induced ROS generation in human monocyte cells (Tsai et al., 2018). In our study, pretreatment with montelukast suppressed arsenic-induced ROS generation. Simultaneously, the activation of AKT-NF-κB signaling is also attenuated by montelukast. NF-κB is a critical transcription factor that is activated by ROS to transactivate the expression of several mesenchymal proteins, such as MMPs, Snail, and Twist. Our results demonstrated that inhibition of NF-κB activation blocked arsenic-induced EMT. These results indicate that montelukast inhibits arsenic-induced total ROS generation, thus inhibiting AKT-NF-κB signaling activation and downstream mesenchymal changes. The scheme summarizing our findings is shown in Figure 6.

Montelukast leads to clinical improvement in asthma-related symptoms both as a monotherapy and in combination with inhaled corticosteroids. Combining oral montelukast with an inhaled corticosteroid, fluticasone, is the most common maintenance therapy for asthmatic children. Glucocorticoids were reported to ameliorate TGF-β-induced EMT in airway epithelial cells (Yang et al., 2017). Treatment with fluticasone decreased TGF-β-induced cell migration and mesenchymal protein (α-SMA, vimentin, and fibronectin) expression. One contrary, the other study has also shown that Fluticasone further enhanced TGF-β-induced fibronectin expression at both the transcriptional and protein levels in fibroblasts (Degen et al., 2009). In our study, fluticasone promoted arsenic-induced cell migration and the expression fibronectin. These results indicated that corticosteroid treatment and the combination of corticosteroid and montelukast may worsen arsenic-induced pulmonary damage.

Taken together, this study revealed novel potential therapeutic agents for treating arsenic-induced pulmonary damage and provides usage guidelines for combination of these agents with corticosteroids. Since our results have shown that montelukast reversed the arsenic-induced EMT in pulmonary epithelial cells, which is believed a mechanism for induction of pulmonary fibrosis, it still needs further *in vivo* studies to validate the therapeutic effect of montelukast for arsenic-induced lung damage. Besides, arsenic exposure-induced airway inflammation is observed in both human (Shih et al., 2019) and rodent (Wang P. et al., 2021; Wang W. et al., 2021; Xiao et al., 2021), and is believed one of the critical mechanisms for arsenic-induced pulmonary damages. For example, arsenic exposure is associated with an increase in eosinophil number in human (Maiti et al., 2012). Notably, montelukast was shown to decrease eosinophil inflammation of asthma patients (Schaper et al., 2011). Therefore, the systemic effects of montelukast on arsenic-induced pulmonary damages *in vivo* is worth for further study.

## Data Availability

The original contributions presented in the study are included in the article/Supplementary Material, further inquiries can be directed to the corresponding author.
