# Supplementary figures and images for: Effects of Montelukast on Arsenic-Induced Epithelial-Mesenchymal Transition and the Role of Reactive Oxygen Species Production in Human Bronchial Epithelial Cells
Source: Front Pharmacol. 2022 Apr 19;13:877125. doi: 10.3389/fphar.2022.877125 (PMC9063880; doi:10.3389/fphar.2022.877125)

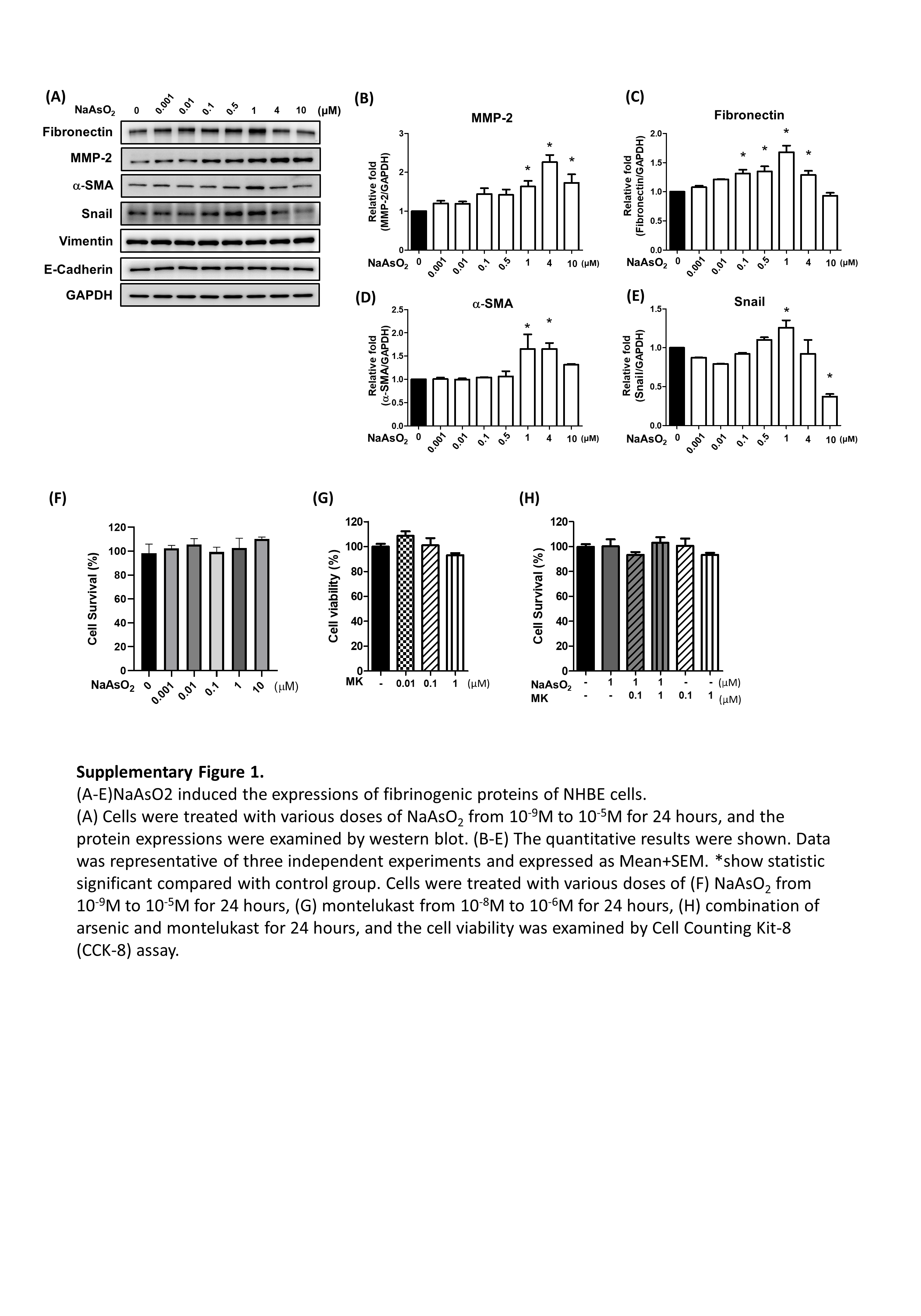

Supplement: Supplementary file 1 [file Image1.tif]
